# *FADS1-FADS2* genetic polymorphisms are associated with fatty acid metabolism through changes in DNA methylation and gene expression

**DOI:** 10.1186/s13148-018-0545-5

**Published:** 2018-08-29

**Authors:** Zhen He, Rong Zhang, Feng Jiang, Hong Zhang, Aihua Zhao, Bo Xu, Li Jin, Tao Wang, Wei Jia, Weiping Jia, Cheng Hu

**Affiliations:** 1 Shanghai Diabetes Institute, Shanghai Key Laboratory of Diabetes Mellitus, Shanghai Clinical Center for Diabetes, Shanghai Jiao Tong University Affiliated Sixth People’s Hospital, 600 Yishan Road, Shanghai, 200233 China; 2 Institute for Metabolic Diseases, Fengxian Central Hospital, The Third School of Clinical Medicine, Southern Medical University, Shanghai, China; 3 Shanghai Key Laboratory of Diabetes Mellitus and Center for Translational Medicine, Shanghai Jiao Tong University Affiliated Sixth People’s Hospital, Shanghai, China; 4 National Clinical Research Center of Kidney Diseases, Jinling Hospital, Nanjing University School of Medicine, Nanjing, China

**Keywords:** DNA methylation, Gene expression, Fatty acids, Genetic markers, Fatty acid desaturase

## Abstract

**Background:**

Genome-wide association studies (GWASs) have shown that genetic variants are important determinants of free fatty acid levels. The mechanisms underlying the associations between genetic variants and free fatty acid levels are incompletely understood. Here, we aimed to identify genetic markers that could influence diverse fatty acid levels in a Chinese population and uncover the molecular mechanisms in terms of DNA methylation and gene expression.

**Results:**

We identified strong associations between single-nucleotide polymorphisms (SNPs) in the fatty acid desaturase (*FADS*) region and multiple polyunsaturated fatty acids. Expression quantitative trait locus (eQTL) analysis of rs174570 on *FADS1* and *FADS2* mRNA levels proved that minor allele of rs174570 was associated with decreased *FADS1* and *FADS2* expression levels (*P* < 0.05). Methylation quantitative trait locus (mQTL) analysis of rs174570 on DNA methylation levels in three selected regions of *FADS* region showed that the methylation levels at four CpG sites in *FADS1*, one CpG site in intragenic region, and three CpG sites in *FADS2* were strongly associated with rs174570 (*P* < 0.05). Then, we demonstrated that methylation levels at three CpG sites in *FADS1* were negatively associated with *FADS1* and *FADS2* expression, while two CpG sites in *FADS2* were positively associated with *FADS1* and *FADS2* expression. Using mediation analysis, we further show that the observed effect of rs174570 on gene expression was tightly correlated with the effect predicted through association with methylation.

**Conclusions:**

Our findings suggest that genetic variants in the *FADS* region are major genetic modifiers that can regulate fatty acid metabolism through epigenetic gene regulation.

**Electronic supplementary material:**

The online version of this article (10.1186/s13148-018-0545-5) contains supplementary material, which is available to authorized users.

## Background

In human physiology, fatty acids have multiple functions. Fatty acids serve as energy sources, substrates for multiple lipid mediators, constituents of cell membranes, and modulators of gene transcription. Fatty acids exist in various forms, including saturated fatty acids (SFAs), trans-fatty acids (TFAs), monounsaturated fatty acids (MUFAs), and polyunsaturated fatty acids (PUFAs) [1, 2]. Numerous prospective epidemiological studies and randomized controlled trials have been conducted to evaluate the involvement of diverse fatty acids in cardiovascular and metabolic diseases [3–5]. High-dose ω-3 fatty acid intake can reduce the risk of noninfarct myocardial fibrosis, while high TFA content in erythrocytes is associated with an elevated risk of coronary heart disease (CHD) [6, 7]. However, the association of ω-6-specific fatty acids with cardiovascular disease is still controversial [8]. Although some earlier experimental studies observed that ω-6-specific PUFA interventions tended to increase CHD risk, a recent meta-analysis based on 13 prospective cohort studies with a total of 310,602 individuals found that higher linoleic acid (LA) intake was associated with a lower risk of CHD events [9–11]. Considering the diverse roles of fatty acids in human diseases, an exploration of the determinants of fatty acid metabolism and concentrations in circulation is needed. Fatty acid levels vary widely according to the individual and to ethnicity, and such differences may be due to environmental factors, especially dietary and/or genetic differences. The mapping of genetic variants that affect serum fatty acid levels may help identify novel genetic markers, uncover disease pathogenesis, and provide pharmaceutical targets for cardiovascular and metabolic diseases.

Genome-wide association studies (GWASs) have identified multiple genetic loci that contribute to inter-individual variations in SFAs, TFAs, MUFAs, and PUFAs in whole plasma, plasma phospholipids, and erythrocyte membrane phospholipids in populations of European ancestry [12–14]. However, the vast majority of the identified loci are noncoding variants, and only a few of these loci have been linked to underlying mechanisms contributing to phenotypic outcome. A validated method for exploring noncoding variation and its influence on complex traits or diseases is linking genetic variants to gene expression or epigenetics in disease-associated tissues [15–17].

Epigenetics can be described as heritable changes that affect gene transcription regardless of the DNA sequence. DNA methylation is an important epigenetic mechanism through which a methyl group, most often a cytosine nucleotide preceding a guanine nucleotide, is added to the DNA sequence [18]. DNA methylation can be affected by both environmental and heritable factors, can affect chromatin structure, and can influence transcription [19].

In this study, we aim to identify genetic markers that could influence diverse fatty acid levels in a Chinese population. Then, by focusing on *FADS1* and *FADS2* (fatty acid desaturase1 and 2), we seek to explain the gene regulatory mechanisms including DNA methylation and gene transcription to help explain the relationships we observed between genomic variation and fatty acid levels.

## Methods

### Participants

The present study represents a subgroup analysis of participants from the Shanghai Obesity Study (SHOS). Detailed study methods have been published previously [20, 21]. Briefly, the SHOS is a community-based, prospective cohort study to investigate the occurrence and development of metabolic syndrome and its related diseases. Beginning in 2009, the SHOS recruited 5000 participants from four communities in Shanghai, China, which included a baseline study as well as 1.5-, 3-, and 5-year follow-up studies. Three hundred twelve adult participants (132 healthy subjects with normal BMI, 107 metabolically healthy participants with overweight or obese state, and 73 metabolically unhealthy participants with overweight or obese state) were recruited in a cross-sectional study for a project focusing on studying the association of serum free fatty acid with metabolic abnormalities. Of these participants, 304 subjects with genotype data available were further selected in our study (mean ± SD age of 48.4 ± 9.7 years and BMI 24.4 ± 3.8 kg/m^2^). A participant flow chart is shown in Additional file 1: Figure S1. Blood samples were collected in the morning after a 12-h fast. EDTA-treated whole blood samples were used for DNA extraction and genotyping with the Infinium Exome BeadChip, and serum samples were used for measuring fatty acid levels.

### Genome-wide genotyping, quality control

Genomic DNA was extracted from peripheral blood leucocytes in whole blood samples. The DNA samples were genotyped using the Infinium Exome-24 v1.0 BeadChip (Illumina, Inc., San Diego, CA, USA), which included a total of 247,870 SNPs. Quality control (QC) was assessed at the individual and SNP levels. First, individuals with high levels of missingness, excess autosomal heterozygosity, high relatedness, ambiguous gender, and ancestry outliers estimated by using ancestry principal component analysis (PCA) were excluded. Next, criteria such as call rate < 98%, significant departures from Hardy–Weinberg equilibrium (*P* < 1 × 10^−6^), significant differences in allele frequency between case and control (*P* > 0.05), not on chromosomes 1–22, and minor allele frequency (MAF) < 1% were applied for excluding SNPs in further analysis. Finally, 32,387 SNPs and 297 individuals were selected for subsequent analysis. The detailed QC results are shown in Additional file 1: Tables S1 and S2; Figures S2, S3, S4 and S5.

### Measurement of serum free fatty acids

All serum samples were stored at − 80 °C until use. The sample preparation procedure for free fatty acid measurements has been described previously [20]. A panel of 42 free fatty acids (FFAs) including 17 SFAs, 12 MUFAs, and 13 PUFAs were subjected to ultra-performance liquid chromatography quadrupole-time-of-flight mass spectrometry (UPLC-QTOFMS) analysis (Waters Corporation, Milford, MA, USA). The ACQUITY-UPLC system was equipped with a binary solvent delivery system and an auto-sampler (Waters Corporation, Milford, USA), coupled to a tandem quadrupole-time-of-flight (Q-TOF) mass spectrometry (Waters Corporation, Milford, USA). A mixture of all the reference standards at an appropriate concentration was prepared and run after every ten serum samples for quality control. The raw data were processed with TargetLynx applications manager version 4.1 (Waters Corporation, Milford, MA, USA). After peak signal detection, standard curve confirmation was needed to calculate the absolute concentration of each FFA. Manual examination and correction were needed to ensure data quality. R version 3.2.1 and SIMCA 13.0.1 software (Umetrics, Sweden) were used for statistical computing and graphics. The relative concentrations of 42 individual fatty acids were expressed as the percentage of total serum fatty acids. Fatty acids with relative concentrations above 0.1% are listed in Table 1. The product-to-precursor ratios were used to estimate the activities of different desaturases as follows: δ-5 desaturase (D5D), arachidonic acid (AA)/dihomo-gamma-linolenic acid (DGLA), and δ-6 desaturase (D6D), gamma-linolenic acid (GLA)/linoleic acid (LA).

**Table 1:** Twenty-two free fatty acids with relative concentrations above 0.1% in 297 subjects

Characteristics	% of total fatty acids
14:0	3.89 ± 1.48
15:0	0.32 ± 0.07
16:0	22.95 ± 2.07
17:0	0.84 ± 0.19
18:0	22.41 ± 4.63
19:0	0.14 ± 0.03
20:0	0.51 ± 0.16
15:0 iso	0.26 ± 0.26
16:0 iso	0.17 ± 0.05
17:0 iso	0.65 ± 0.16
18:0 iso	1.61 ± 0.48
16:1n-7	1.01 ± 0.41
16:1n-9	0.17 ± 0.05
18:1n-9	13.59 ± 3.84
20:1n-9	0.14 ± 0.05
18:2n-6(LA)	20.04 ± 4.76
18:3n-6(GLA)	0.26 ± 0.07
20:2n-6(EDA)	0.32 ± 0.06
20:3n-6(DGLA)	0.36 ± 0.1
20:4n-6(AA)	2.94 ± 0.88
22:4n-6(ADA)	0.33 ± 0.07
22:5n-6	0.19 ± 0.05
18:3n-3(ALA)	0.70 ± 0.27
20:5n-3(EPA)	0.46 ± 0.19
22:5n-3(DPA)	0.58 ± 0.18
22:6n-3(DHA)	4.47 ± 2.00

Data are shown as the mean ± SD. *AA* arachidonic acid, *ADA* adrenic acid, *ALA* α-linolenic acid, *DGLA* dihomo-gamma-linolenic acid, *DHA* docosahexaenoic acid, *DPA* docosapentaenoic acid, *EDA* eicosadienoic acid, *EPA* eicosapentaenoic acid, *GLA* gamma-linolenic acid, *Iso* isomer, *LA* linoleic acid

### Adipose tissue RNA isolation and quantitative PCR

Adipose tissues were previously collected from a metabolic surgery follow-up study [22]. Obese patients from the Department of Endocrinology and Metabolism were recruited in this study, and they received Roux-en-Y gastric bypass surgery. Visceral adipose tissue samples from 42 subjects (age, 43.04 ± 14.90 years; BMI, 34.34 ± 6.37 kg/m^2^) were randomly selected and stored at − 80 °C. RNA from the adipose tissues was extracted with the RNeasy Plus Universal Kit (QIAGEN, Valencia, CA, USA), and 1 μg of RNA was used to synthesize cDNA using the PrimeScript™ RT reagent kit with gDNA Eraser (Takara Bio, Japan). Template cDNAs were diluted 1:4, and the relative expression of *FADS1* and *FADS2* was quantified in triplicate 10-μl reactions by using ABI 7900 Applied Biosystems 7900HT Fast Real-Time PCR System (Applied Biosystems, Foster City, CA, USA) and normalized against the housekeeping gene RPLP0. An assay ID for each gene was assigned as follows: FADS1, Hs00203685_m1; FADS2, Hs00927433_m1; and RPLP0, Hs99999902_m1 (Assays on-demand; Applied Biosystems, Foster City, CA, USA). Each sample was run in triplicates, and the mean value was obtained. Relative quantitative method was used for quantification of mRNA levels.

### Adipose tissue DNA isolation, DNA methylation analysis, and genotyping

Genomic DNA (500 ng) was extracted from the visceral adipose tissues according to the manufacturer’s instructions with the QIAamp DNA Mini kit (Qiagen, Valencia, CA, USA). Bisulfite conversion was performed with the EpiTect Fast DNA Bisulfite kit (Qiagen, Valencia, CA, USA). Based on the CpG densities of the genes, two different methods were used to quantify the DNA methylation levels by matrix-assisted laser desorption ionization-TOF mass spectrometry with a MassARRAY Compact Analyzer (Agena Bioscience, San Diego, CA, USA) [23, 24]. The first method was used for the regions that had high densities of CpGs (CGI1 and CGI2). Specific primers for PCR amplicons covering the majority of the CpG-dense areas in the promoter and in the flanking regions of *FADS1* and *FADS2* were designed with the EpiDesigner tool (http://www.epidesigner.com). After in vitro RNA transcription and subsequent base-specific cleavage reactions, the products were processed in the MassARRAY system. All reactions were performed in duplicate. By comparing the differences in signal intensity between the mass signals derived from the methylated and nonmethylated template DNA, the relative amount of methylation was calculated with EpiTYPER software (Agena Bioscience, San Diego, CA, USA). The signal peaks with overlapping and duplicate units were eliminated in the calculations. The second method was used for the low-density CpG areas. Specific primers for PCR amplicons covering the CpG site were designed with MassARRAY Assay Design software (Agena Bioscience, San Diego, CA, USA). After multiplex amplification and extension of the bisulfite-treated DNA in a single reaction and processing in the MassARRAY system, the methylation data of the individual CpG sites were generated with the TyperAnalyzer software (Agena Bioscience, San Diego, CA, USA). Duplicate measurements with differences equal to or greater than 10% were discarded.

rs174570 was selected from the seven SNPs (rs102275, rs174546, rs174547, rs174550, rs174570, rs1535, and rs174583) that were significantly associated with FFA metabolism and genotyped in DNA samples from the visceral adipose tissues of 42 participants using 3500 Genetic Analyzer (Applied Biosystem, Foster City, CA, USA).

### Statistical analysis

Data are presented as the mean ± SD or median (interquartile range). Variables with skewed distribution were appropriately transformed before analyses. The Hardy–Weinberg equilibrium was tested before the association analysis, and all seven SNPs were in accordance with the Hardy–Weinberg equilibrium (all *P* > 0.05). Linkage disequilibrium analysis was performed in Haploview 4.2, relying on all individuals in the population, and was assessed with *D*^′^ and *r*^2^ values. For each fatty acid, the distribution of *P* values was evaluated using a quantile–quantile plot. The Q–Q plots for the nine most studied PUFAs are shown in Additional file 1: Figure S5. To explore the possible molecular mechanism of DNA methylation and gene expression in gene–nutrition interaction, we further conducted a series of pre-specified analyses based on biological plausibility and the literature. Linear regression analysis was used to estimate the associations of genotypes with fatty acid levels and mRNA expression with adjustments for age, sex, and BMI with PLINK 1.07. The effects of rs174570 on the DNA methylation levels of the CpG sites were analyzed using the Kruskal–Wallis *H* test. Pearson’s correlation analysis was applied to test the correlation between mRNA expression and the methylation level.

Mediation analysis was performed to explore the relationship between the predictor and outcome variables and was achieved with the PROCESS procedure implemented in SPSS20.0 [25]. Bootstrap analyses (with 5000 resamples) were performed to test the significance of the differences in the predictor–outcome associations after adding a mediator as a covariate. A mediation effect was considered significant if 0 was not included in the bootstrap confidence interval. In our study, we established models relating rs174570 as a predictor to FADS1 and FADS2 gene expression outcome including DNA methylation as a covariate. Analyses were adjusted for age, sex, and BMI. The model assumes no unmeasured confounding or modification effect between the included variables.

Statistical analyses were carried out using SAS 9.3 (SAS Institute, Cary, NC, USA) unless specified otherwise. Statistical significance was defined as a two-tailed *P* < 0.05.

## Results

### Identification of SNPs involved in fatty acid metabolism

**Fig. 1:**
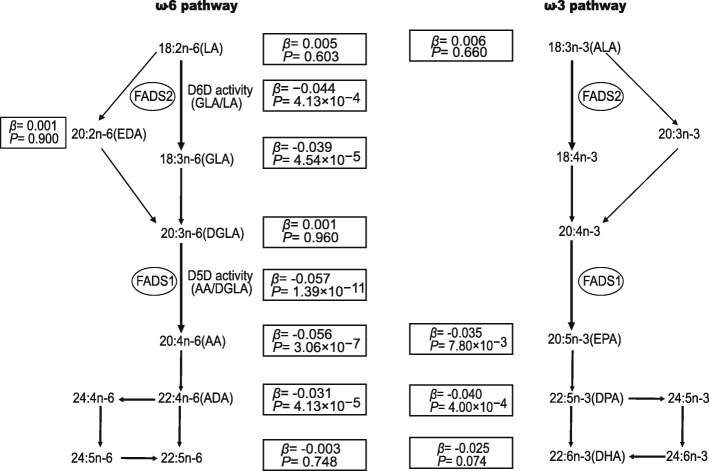
Biosynthetic pathway of ω-3 and ω-6 long-chain polyunsaturated fatty acids (LC-PUFAs) from essential fatty acids and the associations of rs174570 with PUFAs in the ω-3 and ω-6 pathways. LA and ALA are essential fatty acids that must be obtained from the diet and are converted to long-chain polyunsaturated fatty acids. LC-PUFAs are synthesized via a series of actions of elongases and desaturases. DPA and DHA are mainly obtained from the diet or indirectly obtained by de novo synthesis from essential fatty acids. Linear regression analysis with adjustments for age, sex, and BMI was performed. The associations between rs174570 and the PUFAs in the ω-3 and ω-6 pathways are shown with *β* and *P* values in the black boxes. LA, linoleic acid; GLA, gamma-linolenic acid; DGLA, dihomo-gamma-linolenic acid; EDA, eicosadienoic acid; AA, arachidonic acid; ADA, adrenic acid; ALA, α-linolenic acid; EPA, eicosapentaenoic acid; DPA, docosapentaenoic acid; DHA, docosahexaenoic acid; FADS, fatty acid desaturase

**Fig. 2:**
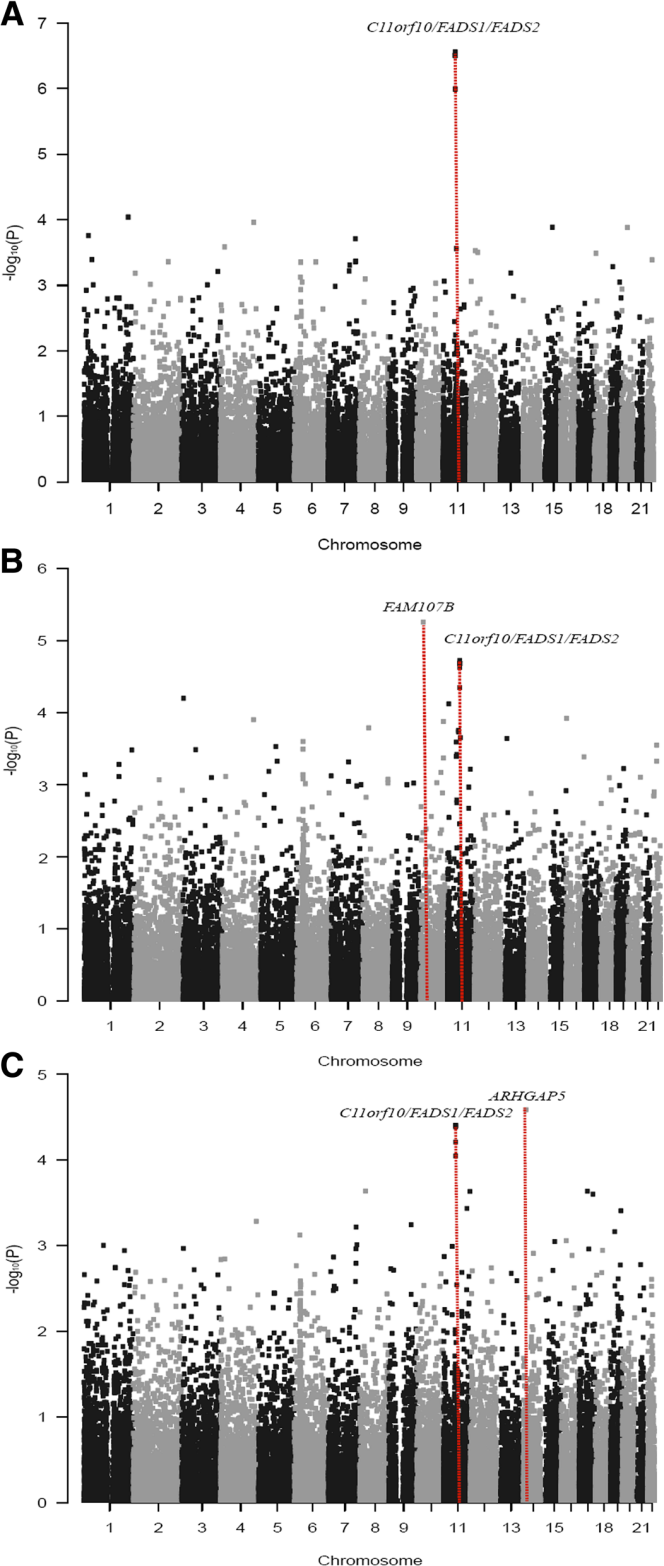
Genome-wide Manhattan plot for arachidonic acid (**a**), gamma-linolenic acid (**b**), and adrenic acid (**c**). Plots of –log_10_(*P*) values for associations tested by linear regression analysis with adjustments for age, sex, and BMI under an additive genetic model against fatty acid levels across the entire autosomal genome are shown. The red dots at each locus indicate the signals with *P* < 10^−4^ detected in the genome-wide analysis. A total of 32,387 SNPs was used to generate the plots. FADS, fatty acid desaturase; ARHGAP5, P190-B RhoGAP; FAM107B, family with sequence similarity 107 member B

To explore the potential effects of genetic variants on fatty acid metabolism, we tested the association of 32,387 SNPs that passed the QC standards with 42 fatty acids in 297 subjects. A total of 317 linear mixed models of SNP–fatty acid combinations reached significant *P* values of less than 10^−4^. A cluster of seven SNPs in tight linkage disequilibrium (rs174546, rs174547, rs174550, rs174570, rs1535, rs174583, and rs102275) in one locus that was on chromosome 11, containing *FADS1*, *FADS2*, and *C11orf10*, was significantly associated with multiple serum fatty acid levels. Characteristics of the seven SNPs analyzed in the *C11orf10*/*FADS1*/*FADS2* gene cluster were listed (Additional file 1: Table S3). Based on the high linkage disequilibrium of these seven SNPs (*r*^2^ = 0.98–1, Additional file 1: Figure S6), rs174570 was selected in the next analysis due to differences in the allele frequencies among different ethnicities from the International HapMap project. We showed that the minor allele (T) of rs174570 was associated with lower levels of GLA, AA, and adrenic acid (ADA) in the ω-6 pathway and with lower levels of docosapentaenoic acid (DPA) and eicosapentaenoic acid (EPA) in the ω-3 pathway (all *P* < 0.05). GLA was the product of the D6D-catalyzed reaction, while AA and ADA in the ω-6 pathway and DPA and EPA in the ω-3 pathway served as D5D products. However, no associations were evident between rs174570 and other fatty acids (Fig. 1 and Additional file 1: Table S4). The Manhattan plots in Fig. 2 show the genome-wide associations of AA, GLA, and ADA. Moreover, using ratios of ω -6 PUFAs as proxies of desaturase activities, we confirmed that the minor allele of rs174570 was associated with lower D5D and D6D activities (Fig. 1).

### Expression quantitative trait locus (eQTL) analysis of rs174570 on gene expression

**Fig. 3:**
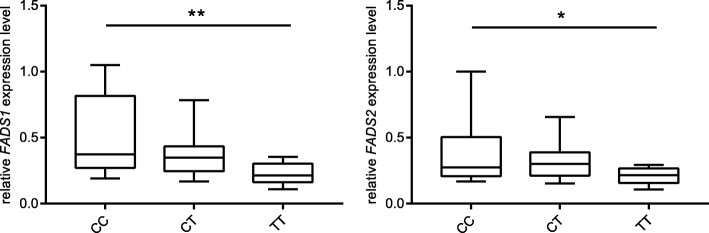
The relative *FADS1* and *FADS2* mRNA levels of the CC (*n* = 13), CT (*n* = 17), and TT (*n* = 12) carriers at rs174570 were quantified by qPCR with *RPLP0* as the reference gene. For each box, the median is indicated by the band inside the box. The first and third quartiles are shown at the bottom and top of the box, respectively. **P < 0.01, *P < 0.05 tested by linear regression analysis with adjustments for age, sex, and BMI. FADS, fatty acid desaturase; RPLP0, ribosomal protein lateral stalk subunit P0

To better understand the potential functional roles of the significant genetic variant, we performed eQTL analysis with adjustments for age, sex, and BMI to test the association of rs174570 with the mRNA levels of *FADS1* and *FADS2* in the visceral adipose tissues of 42 participants. As shown in Fig. 3, the *FADS1* and *FADS2* mRNA levels were significantly reduced in the TT carriers compared with the CC and TT carriers (*P* = 0.001, *β* ± SE = − 0.142 ± 0.040 for *FADS1*; *P* = 0.016, *β* ± SE = − 0.094 ± 0.037 for *FADS2*). These data suggest that rs174570 is a strong eQTL and that the observed decrease in desaturase activity is mediated by variant-dependent inhibition of *FADS1* and *FADS2* expression.

### Methylation quantitative trait locus (mQTL) analysis of rs174570 on DNA methylation levels

**Fig. 4:**
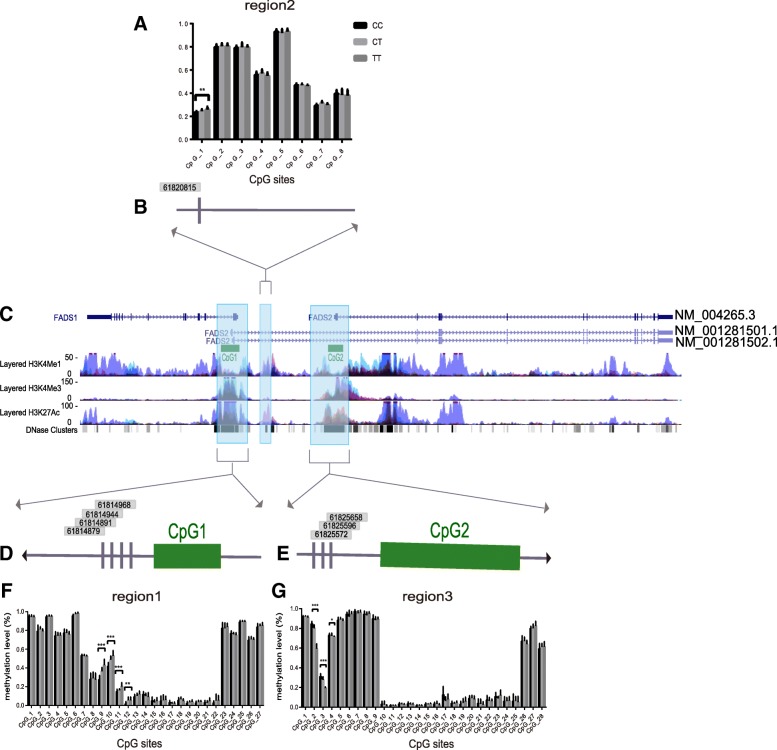
*FADS1* and *FADS2* gene structure and the methylation patterns of the three regions (region1, region2, and region3). (C) The three regions are indicated with blue shaded boxes. *FADS1* and *FADS2* are located head to head on chromosome 11. Two CpG islands (CGI1 and CGI2) are depicted as green solid boxes on the left and right sides, covering the first exons of *FADS1* and *FADS2*, respectively (GRCh38/hg38 Assembly). The H3K4Me1, H3K4Me3, and H3K27Ac marks on 7 cell lines from ENCODE are displayed as colored overlaid histograms. DNase Hypersensitivity 1 regions are displayed as gray to black solid boxes (from less to more open chromatin conformation). NM_001281501.1, NM_001281502.1, and NM_004265.3 refer to different transcript variants due to alternative splicing (NCBI Reference Sequence). (A, F, and G) DNA methylation levels of the CpG sites of the CC (*n* = 13), CT (*n* = 17), and TT (*n* = 12) carriers in the three regions. A total of 27 CpG sites in region1, 8 CpG sites in region2, and 28 CpG sites in region3 are shown in the bar charts. (B, D, and E) The genomic locations of the CpG sites that were significantly associated with rs174570 are shown. The arrow represents the direction of the gene, and the filled rectangles represent the CpG sites. The specific position of each CpG site on the chromosome is marked in a gray shaded box. ***P < 0.001, **P < 0.01, *P < 0.05. FADS, fatty acid desaturase

To examine whether genetic variation was associated with DNA methylation levels in the human visceral fatty tissues, three regions (region1, region2, and region3) were selected for mQTL analysis. Region1 and region3 were selected due to the inclusion of two CpG islands (CGI1 and CGI2, respectively), each covering the first exons of *FADS1* and *FADS2*, respectively, with potential promoter activity (Fig. 4). Region2 with putative enhancer activity was selected to validate the results of previous studies conducted by Floyd H. Chilton et al. [26, 27]. These authors showed that the DNA methylation levels of multiple CpG sites in this region were associated with rs174537. A total of 27 CpG sites in region1 covering 4.2 kb, 8 CpG sites in region2 covering 1 kb, and 28 CpG sites in region3 covering 5.2 kb passed QC and were selected for further analysis. As shown in Fig. 4 (F, G), distinct patterns of the methylation levels were observed for CpG sites in the genome, with lower methylation levels for CpG sites in potential promoters than in other regions. Then, we compared the DNA methylation levels of the different groups according to the rs174570 genotype. The methylation levels of CpG sites CpG_9, CpG_10, CpG_11, and CpG_12 in region1 and CpG_1 in region2 were significantly higher in the TT genotype group than in the CC and CT genotype groups (*P* < 0.05), while the CpG methylation levels of sites CpG_2, CpG_3, and CpG_4 in region3 were significantly lower in the TT group than in the CC and CT groups (*P* < 0.05). However, the methylation levels at the other sites, including four CpG sites (chr11:61587979, 61588092, 61588096, and 61588188 based on genome build GRCh37/hg19) that were found to be associated with rs174537 in another study, did not significantly differ (*P* > 0.05 for all). Consistent with the previous data suggesting that clusters of adjacent CpG sites are co-regulated, we showed that rs174570 had a directionally consistent effect on methylation levels at four CpG sites in *FADS1* and three CpG sites in *FADS2*. Table 2 provides an overview of the eight CpG sites, summarizing the location information and sequences around the CpG sites in the genome.

**Table 2:** Specific genomic information of CpG sites shown to be associated with the rs174570

Region	CpG site	Location	Sequence
Region1	CpG_9	Chr11: 61814879	GAAAGACCCGCAAGAAGGGA [CG]GAAGTCTCATAGCCCTGAGA
	CpG_10	Chr11: 61814891	AAGGGACTTATTGAAAGACC [CG]CAAGAAGGGACGGAAGTCTC
	CpG_11	Chr11: 61814944	CGTAGGGAAGTCTTCCTCTT [CG]TGGTTTTTGGAGAACCCTAG
	CpG_12	Chr11: 61814968	AACGCAGAAGTGCCCCAGTT [CG]GACGTAGGGAAGTCTTCCTC
Region2	CpG_1	Chr11: 61820815	CTTTGCCTCCTGGGTTCAAG [CG]ATTCTCCTGCCTCACCCCCA
Region3	CpG_2	Chr11: 61825572	AGGTTGCAGTGAGCTGAGAT [CG]CACCACTGCACTCCAGCCTG
	CpG_3	Chr11: 61825596	CCACTGCACTCCAGCCTGGG [CG]ACAGAGTGAGACCCTGTCTC
	CpG_4	Chr11: 61825658	GAAAAAGCCCTTTGGGAGGC [CG]AGGCAGGTGGATCACGAGGT

Region1, region2, and region3 indicate the selected regions used for DNA methylation analysis; sequence indicates 20 bp upstream and downstream of the CpG site in the genome (GRCh38/hg38 Assembly). *Chr* chromosome

### Correlation analysis of DNA methylation levels with gene expression

Then, we tested the correlations between gene expression and the DNA methylation levels of the eight CpG sites that were confirmed to be significantly associated with rs174570. We found that three CpG sites in region1 (CpG_9, CpG_10, and CpG_11) and two CpG sites (CpG_2 and CpG_3) in region3 were significantly associated with both *FADS1* and *FADS2* expression. However, we did not observe a significant correlation between CpG_1 in region2 and gene expression. Notably, the *FADS1* and *FADS2* expression levels decreased with the increasing methylation levels of the three CpG sites in region1, while the *FADS1* and *FADS2* expression levels increased with the increasing methylation levels of the two CpG sites in region3 (Table 3).

**Table 3:** Correlations of DNA methylation levels with *FADS1* and *FADS2* gene expression

Region	CpG site	*FADS1* mRNA levels		*FADS2* mRNA levels	
		*r*	*P* ^1^	*r*	*P* ^2^
Region1	CpG_9	− 0.501	< 0.001	− 0.363	0.020
	CpG_10	− 0.527	< 0.001	− 0.420	0.006
	CpG_11	− 0.428	0.005	− 0.419	0.006
	CpG_12	− 0.157	0.328	− 0.085	0.600
Region2	CpG_1	− 0.134	0.397	− 0.118	0.456
Region3	CpG_2	0.548	< 0.001	0.478	0.001
	CpG_3	0.565	< 0.001	0.459	0.003
	CpG_4	0.200	0.2062	0.235	0.135

*P*^1^ and *P*^2^ separately indicate the correlation of DNA methylation of CpG site with *FADS1* or *FADS2* gene expression tested by Pearson’s correlation analysis. *FADS* fatty acid desaturase

### Mediation analysis reveals that DNA methylation potentially mediates the genetic impact on mRNA expression

To investigate whether DNA methylation functioned as a mediator for the observed association between genotype and phenotype (gene expression), mediation analysis was conducted with the following two statistical criteria. First, the effect value of the original association was reduced with the addition of a potential mediator to the model. Second, the differences before and after the addition of the potential mediator were statistically significantly tested with a bootstrap analysis (*P* < 0.05). Bootstrap analysis was performed with DNA methylation levels at five CpG sites (CpG_9, CpG_10, and CpG_11 in region1 and CpG_2 and CpG_3 in region3) as the mediator, the genotyping of rs174570 as the independent variable, and the FADS1 or FADS2 expression levels as the dependent variable. Based on the two statistical criteria, we found that the differences before and after the addition of DNA methylation levels at CpG_9, CpG_10, CpG_2, or CpG_3 were significant for *FADS1* gene expression. However, only the difference in the regression coefficient with and without the addition of the CpG_2 methylation level was significant for *FADS2* gene expression (Table 4). These data demonstrate that the DNA methylation levels of four CpG sites mediated the effect of rs174570 on *FADS1* gene expression, while only DNA methylation of one CpG site mediated the effect of rs174570 on *FADS2* gene expression.

**Table 4:** Mediation effects of DNA methylation levels on the association between rs174570 and gene expression

Outcome	Total effect		Model	Direct effect		Bootstrap analysis	
	*β* ± SE	*P* ^1^		*β* ± SE	*P* ^2^	Boot LLCI	Boot ULCI
*FADS1* mRNA level	− 0.142 ± 0.040	0.001	Adjusted for CpG_9	− 0.019 ± 0.068	0.782	*− 0.264*	*− 0.023*
			Adjusted for CpG_10	− 0.066 ± 0.055	0.235	*− 0.173*	*− 0.014*
			Adjusted for CpG_11	− 0.115 ± 0.054	0.042	− 0.090	0.068
			Adjusted for CpG_2	0.002 ± 0.080	0.976	*− 0.284*	*− 0.005*
			Adjusted for CpG_3	0.043 ± 0.075	0.572	*− 0.295*	*− 0.047*
*FADS2* mRNA level	− 0.094 ± 0.037	0.016	Adjusted for CpG_9	0.002 ± 0.063	0.977	− 0.217	0.004
			Adjusted for CpG_10	− 0.038 ± 0.519	0.472	− 0.162	0.004
			Adjusted for CpG_11	− 0.055 ± 0.050	0.275	− 0.103	0.032
			Adjusted for CpG_2	0.042 ± 0.074	0.571	*− 0.265*	*− 0.008*
			Adjusted for CpG_3	0.066 ± 0.070	0.350	− 0.243	0.005

*P*^1^ represents the total effect of rs174570 on *FADS1* or *FADS2* gene expression without adjustment for DNA methylation; *P*^2^ represents the effect of rs174570 on *FADS1* or *FADS2* gene expression controlling for DNA methylation. A mediation effect was considered significant when 0 was not included in the bootstrap confidence interval. The characters shown in italics refer to a significant mediation effect. *Boot LLCI*, Boot low limit confidence interval; *Boot ULCI*, Boot upper limit confidence interval; *FADS*, fatty acid desaturase

## Discussion

In this exome-wide association study of fatty acid levels in a Chinese population, we identified several strong signals on the *FADS* region and confirmed the associations of polymorphisms in FADS gene cluster with decreased GLA, AA, ADA, EPA, and DPA, but the strongest associations were with D5D activity in the ω-6 pathway. Long-chain ω-3 and ω-6 PUFAs, including AA, EPA, and DPA, are products of the ω-3 and ω-6 pathways that are converted from essential fatty acids (LA and ALA) by fatty acid desaturases and elongases (Fig. 1). AA is a precursor of multiple pro-inflammatory factors, including prostaglandin E2 and thromboxane A2 which have a harmful impact on the development of cardiovascular disease, while EPA improves biomarkers of inflammation and has a protective effect [28–30]. Thus, major allele (C) carriers with greater desaturase activity should have both pro-inflammatory and anti-inflammatory outcomes. Due to competition between ω-3 and ω-6 PUFAs for the same desaturases and due to the higher levels of LA intake than ALA intake in the modern Western diet, individuals with the CC genotype may be vulnerable to inflammatory disorders when consuming a diet that is rich in ω-6 fatty acids. Thus, CC carriers should consume a diet that is rich in ALA, which may reduce the synthesis of ω-6 PUFAs, or adopt a diet containing more EPA and docosahexaenoic acid (DHA), which directly balance AA.

Although numerous studies have focused on how polymorphisms in *FADS1* and *FADS2* alter fatty acid profiles or desaturase activity, few have investigated whether these polymorphisms regulate fatty acid metabolism by altering gene expression and epigenetic modifications in human tissues. By performing the eQTL and mQTL analyses in this study, we showed that rs174570 was associated with gene expression and methylation levels at multiple local CpG sites in the visceral adipose tissue. These results suggest that both DNA methylation and gene transcription may account for the association between genetic variants in the *FADS* region and different PUFA levels.

DNA methylation has long been considered an important regulator of gene expression, and DNA methylation levels of CpG sites in different regions may have different effects on gene expression [31, 32]. In our study, we observed a negative correlation between DNA methylation and gene expression in region1 with putative promoter activity. This is consistent with a previous concept that DNA methylation in promoter regions may suppress gene expression by affecting the physical access of transcription factors. However, the underlying mechanism has not been clarified. We used publicly available UCSC annotations (GRCh38/hg38 Assembly) to evaluate histone modification and transcription factor binding sites. The results from UCSC show that region1 is an active promoter site, as indicated by the tri-methylation of lysine 4 of the H3 histone protein. There are several transcription factor binding sites near our highlighted CpGs in region1, one of which, namely, SP1, has been shown to be involved in the regulation of *FADS1* expression by Pan et al. [33]. This study demonstrated that the minor allele of rs174557, which was approximately 1 kb away from our highlighted CpGs, was associated with the activating complex of SP1 and sterol regulatory element-binding protein (SREBP1c), together decreasing *FADS1* expression level. SREBP1c is an important transcription factor that regulates *FADS* expression and has been investigated intensively [34, 35]. Thus, future studies are needed to determine whether SREBP1c or other factors play roles in the effect of DNA methylation on *FADS* expression. In contrast to methylation in promoter regions, gene-body methylation is not necessarily associated with repression. One major explanation may link DNA methylation to alternative splicing, and another possible mechanism may be that DNA methylation promotes more efficient transcriptional elongation of upstream promoters while simultaneously repressing spurious transcription from intragenic promoters [36–39]. Our data suggested a positive correlation of methylation levels at CpG sites in region3 with *FADS1* and *FADS2* expression. These data are consistent with a previous genome-wide study showing that 35.4% of the associations between gene expression and DNA methylation levels at intragenic CpGs in human pancreatic islets are positive. Notably, three transcript variants of *FADS2*, including NM_004265.3, NM_001281501.1, and NM_001281502.1, exist. The first transcript is the traditional isoform and is located downstream of the last two transcripts (Fig. 4). Thus, *FADS2* may have at least two TSSs, and the downstream start sites are within the “bodies” of the transcriptional units of the upstream promoters, providing a foundation for what we observed in our study.

In addition, we note that other studies conducted by Floyd H. Chilton et al. [26, 27] have focused on exploring the effects of genetic variation on DNA methylation and found a significant association between rs174537 and the methylation statuses of multiple CpG sites between *FADS1* and *FADS*. In our study, eight CpG sites in this region were selected for DNA methylation analysis and the methylation level of only one CpG site (CpG_1 in region2) was associated with rs174570. However, Floyd H. Chilton et al. [26, 27] did not find an association between rs174537 and the methylation level of CpG_1. The reason for the lack of association between rs174570 and the methylation levels at the CpG sites identified in the other study may have been that the different SNPs from different races were used in the two studies (rs174537 from European Americans and African Americans in the other study versus rs174570 from a Chinese population in our study). Furthermore, the authors in the other study did not identify the CpG sites that were shown to be statistically significant in our study. Indeed, the Illumina HumanMethylation450 array covers less than 2% of the estimated 30 million CpG sites in the human genome [40]. Among the 63 CpG sites analyzed in our study, only one CpG site (CpG_16 in region3) was assessed with the 450K array. These results suggest that studies focusing on methylation levels of CpG sites in potential regulatory elements are potent complement for global DNA methylation studies to identify novel CpG sites that are associated with related phenotypes. In addition, we did not observe a correlation between the DNA methylation level of CpG_1 in region2 and gene expression in our study, which was not shown in studies conducted by Floyd H. Chilton et al. [26, 27]. Thus, further studies in different populations are needed to elucidate whether the DNA methylation levels of CpG sites in this region contribute to variations in gene expression.

We performed mediation analysis to investigate the potential mediator effect of methylation on the relationship between rs174570 and gene expression. Mediation analysis has been used in several studies to evaluate the mediation effect of DNA methylation levels on interactions between predictor and outcome variables [41, 42]. In this study, we identified DNA methylation levels at several CpG sites as mediators by applying this approach. However, not all significant CpG sites that were associated with both rs174570 and gene expression mediated the genetic effect on expression. These results suggest that DNA methylation is not the only pathway explaining the association between rs174570 and gene expression.

In addition to the observation that genetic variants of *FADS* cluster exerted an effect on gene expression by changing DNA methylation levels, substantial evidence suggests that dietary supplementation can modulate DNA methylation status of *FADS* gene cluster that affect gene transcription [43, 44]. These findings suggest that epigenetics may serve as a common pathway mediating both the genetic and environmental effect on gene function and as an underlying mechanism for gene–environment interaction, which are now recognized as an important implication for complex phenotypes [45, 46]. It is possible that genetically controlled methylation sites are sensitive to environmental changes and can serve as mediators of disease susceptibility [47]. Thus, understanding the impact of polymorphisms on the epigenetics provides instruction on early dietary interventions to modify long-term disease risk. However, it is still not clear whether DNA methylation status of *FADS* gene cluster conveys the information from gene–environment interactions to specific disease disorders. Clinical trials on investigating the effect of nutrition intake on DNA methylation with the consideration of genotypes are urgently needed.

The limitation of our study was that we did not consider the effect of dietary fatty acids on DNA methylation. There is growing evidence that complex interactions among food components and genetic factors lead to a dynamic modification of DNA methylation that controls the phenotype [48, 49]. However, we did not have the relevant data for dietary fatty acid intake in this study, and thus, we could not evaluate the effects of dietary fatty acids on DNA methylation.

## Conclusions

Overall, our results support that DNA methylation and gene regulation have important implications for the interpretation of the effects of noncoding sequence variations on phenotypic outcomes. Further studies are needed to investigate how rs174570 in the intronic region of *FADS2* influences DNA methylation and to uncover the mechanisms underlying the specific effects of DNA methylation on *FADS1* and *FADS2* expression.

## Additional file

Additional file 1: Supplementary data. **Table S1.** Quality control (QC) at the individual level. **Table S2.** Quality control (QC) at the SNP level. **Table S3.** Characteristics of the seven SNPs analyzed in the *C11orf10 /FADS1/FADS2* gene cluster. **Table S4.** Associations between rs174570 and free fatty acid levels.** Figure S1.** The participant flow chart in this study. SHOS, Shanghai Obesity Study; MH-NW, metabolically healthy participants with normal BMI; MHO, metabolically healthy participants with overweight or obese state; metabolically unhealthy participants with overweight or obese state. **Figure S2.** The proportion of missing genotypes against the heterozygosity rate is plotted. The overall call rate is greater than 0.98, and the heterozygosity rate is also considered to remove tainted and inbred samples. **Figure S3.** The detection of identity by descent segments in close relative pairs using unphased dense SNP data shows pairwise relatedness in the cohort. MZ indicates monozygotic twins, FS indicates full-siblings, PO indicates parent-offspring, and HS indicates half-siblings. **Figure S4.** Individuals were plotted based on the first two eigenvectors produced by principal component analysis (PCA), (A) based on the genotype data of 11 populations from HapMap (African ancestry in southwestern USA [ASW], Utah residents with northern and western European ancestry from the CEPH collection [CEU], Han Chinese in Beijing, China [CHB], Chinese in metropolitan Denver, Colorado [CHD], Gujarati Indians in Houston, Texas [GIH], Japanese in Tokyo, Japan [JPT], Luhya in Webuye, Kenya [LWK], Mexican ancestry in Los Angeles, California [MEX], Maasai in Kinyawa, Kenya [MKK], Tuscan in Italy [TSI], and Yoruban in Ibadan, Nigeria [YRI]) and from the cohort of the present study (HAN). (B) Based on the genotype data of our cohort in the present study. **Figure S5.** Quantile-quantile plots for (A) linoleic acid, (B) a-linolenic acid (C) gamma-linolenic acid, (D) dihomo-gamma-linolenic acid, (E) arachidonic acid, (F) eicosapentaenoic acid, (G) adrenic acid, (H) docosapentaenoic acid, and (I) docosahexaenoic acid. The observed *P*-values of the indicated SNPs are plotted against the theoretical distributions of the expected *P*-values. A total of 32,387 SNPs were used to generate each plot. **Figure S6.** Linkage disequilibrium maps for single nucleotide polymorphisms (SNPs) genotyped in FADS and the flanking regions. Color schemes are based on the D’ value in the Haploview software. The shades of red show the strength of the pairwise linkage disequilibrium, and the numbers represent the D’ values expressed as percentages. (PDF 652 kb)

## Abbreviations

AA: Arachidonic acid
ADA: Adrenic acid
D5D: δ-5 Desaturase
D6D: δ-6 Desaturase
DGLA: Dihomo-gamma-linolenic acid
DPA: Docosapentaenoic acid
eQTL: Expression quantitative trait locus
FADS: Fatty acid desaturase
GLA: Gamma-linolenic acid
GWASs: Genome-wide association studies
LA: Linoleic acid
mQTL: Methylation quantitative trait locus
MUFAs: Monounsaturated fatty acids
QC: Quality control
SHOS: Shanghai Obesity Study
SNPs: Single-nucleotide polymorphisms
SREBP1c: Sterol regulatory element-binding protein
TFAs: Trans-fatty acids
