# Supplementary material for: FADS1-FADS2 genetic polymorphisms are associated with fatty acid metabolism through changes in DNA methylation and gene expression
Source: Clin Epigenetics. 2018 Aug 29;10:113. doi: 10.1186/s13148-018-0545-5 (PMC6114248; doi:10.1186/s13148-018-0545-5)

Supplementary data

Supplemental Table 1. Quality control (QC) at the individual level.

	Participants
Number of subject before QC	304
Exclusion criteria	
Overall call rate < 0.98	0
Abnormal heterozygous genotype ratio	5
Relatedness	2
Gender problem	0
Problem of population stratification	0
Number of subjects after QC	297

Supplemental Table 2. Quality control (QC) at the SNP level.

	SNPs
Number of SNPs before QC	247,870
Exclusion criteria:	
SNP call rate is < 98%	130
SNPs without HWE ($P < 1\times10^{-6}$)	112
SNPs with different call rates between the case and control (>0.05)	0
Number of SNPs not on chromosomes 1-22	23,602
SNPs with MAF < 1%	210,013
Number of SNPs on chromosomes 1-22 after QC	32,387

SNP, single nucleotide polymorphism; HWE, Hardy-Weinberg equilibrium; MAF, minor allele frequency.

Supplemental Table 3. Characteristics of the seven SNPs analyzed in the *C11orf10*/*FADS1*/*FADS2* gene cluster.

SNP	Gene	Chr: position	Functional Region	Minor Allele	MAF
rs102275	C11orf10	11:61790331	intron	C	0.399
rs174546	FADS1	11:61802358	3’UTR	T	0.3973
rs174547	FADS1	11:61803311	intron	C	0.3973
rs174550	FADS1	11:61804006	intron	C	0.3973
rs174570	FADS2	11:61829740	intron	T	0.3956
rs1535	FADS2	11:61830500	intron	C	0.3973
rs174583	FADS2	11:61842278	intron	T	0.4007

SNP, single nucleotide polymorphism; FADS, fatty acid desaturase; Chr: position, position in chromosome (GRCh38/hg38 Assembly). MAF, minor allele frequency.

Supplemental Table 4. Associations between rs174570 and free fatty acid levels.

Subgroup	Free Fatty acids	β	SE	P
SFAs	8:0	-0.0466	0.05043	0.3562
	10:0	-0.01827	0.04697	0.6975
	12:0	0.02149	0.03659	0.5574
	14:0	-0.007078	0.01271	0.5779
	15:0	0.0003694	0.007929	0.9629
	16:0	0.002477	0.003263	0.4483
	17:0	0.002885	0.008117	0.7225
	18:0	0.003265	0.007613	0.6683
	19:0	0.005099	0.00898	0.5706
	20:0	0.005873	0.01131	0.6038
	22:0	0.0004093	0.01168	0.9721
	24:0	0.006643	0.02029	0.7437
	14:0 iso	0.009707	0.01175	0.4093
	15:0 iso	-0.009003	0.01187	0.4488
	16:0 iso	-0.002186	0.01304	0.867
	17:0 iso	0.001407	0.009419	0.8814
	18:0 iso	0.008904	0.01116	0.4254
MUFAs	14:1n-5	0.004764	0.01696	0.779
	Trans-14:1n-5	0.008139	0.02365	0.7309
	16:1n-7	0.007515	0.01461	0.6074
	Trans-16:1n-7	-0.01204	0.02142	0.5745
	16:1n-9	0.006793	0.01116	0.5433
	17:1n-7	0.01898	0.01421	0.1828
	18:1n-9	0.01536	0.01185	0.1959
	Trans-18:1n-9	0.01421	0.01514	0.3489
	19:1n-9	0.01255	0.02451	0.609
	20:1n-9	0.007155	0.0117	0.5415
	22:1n-9	-0.01589	0.02415	0.5112
	24:1n-9	0.004098	0.01056	0.6984
PUFAs	22:2n-6	0.01874	0.008876	0.06559
	22:3n-3	0.01132	0.01088	0.2993

SFAs, saturated fatty acids; MUFAs, monounsaturated fatty acids; PUFAs, polyunsaturated fatty acids.

Supplemental Figure 1. The participant flow chart in this study. SHOS, Shanghai Obesity Study; MH-NW, metabolically healthy participants with normal BMI; MHO, metabolically healthy participants with overweight or obese state; metabolically unhealthy participants with overweight or obese state.

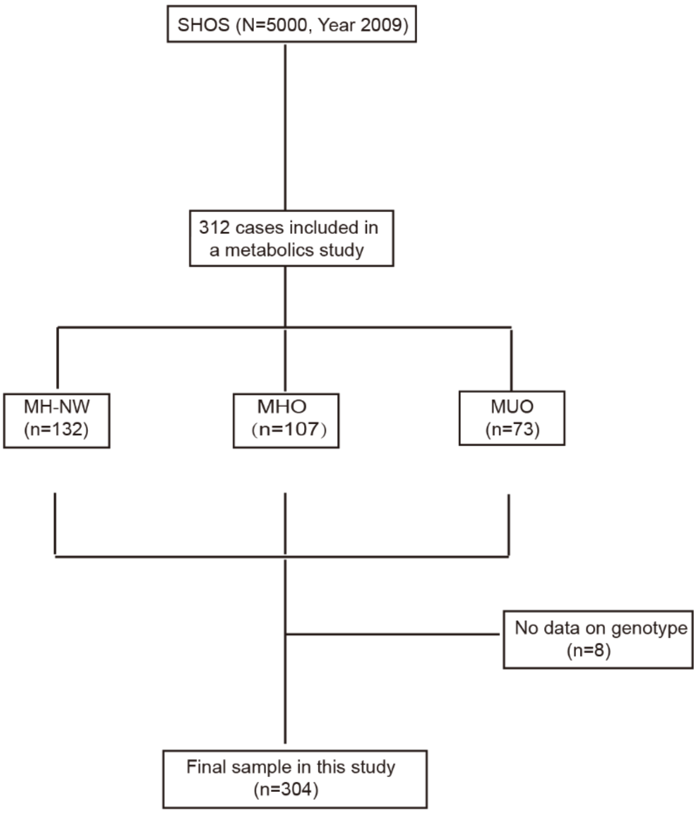

Supplemental Figure 2. The proportion of missing genotypes against the heterozygosity rate is plotted. The overall call rate is greater than 0.98, and the heterozygosity rate is also considered to remove tainted and inbred samples.

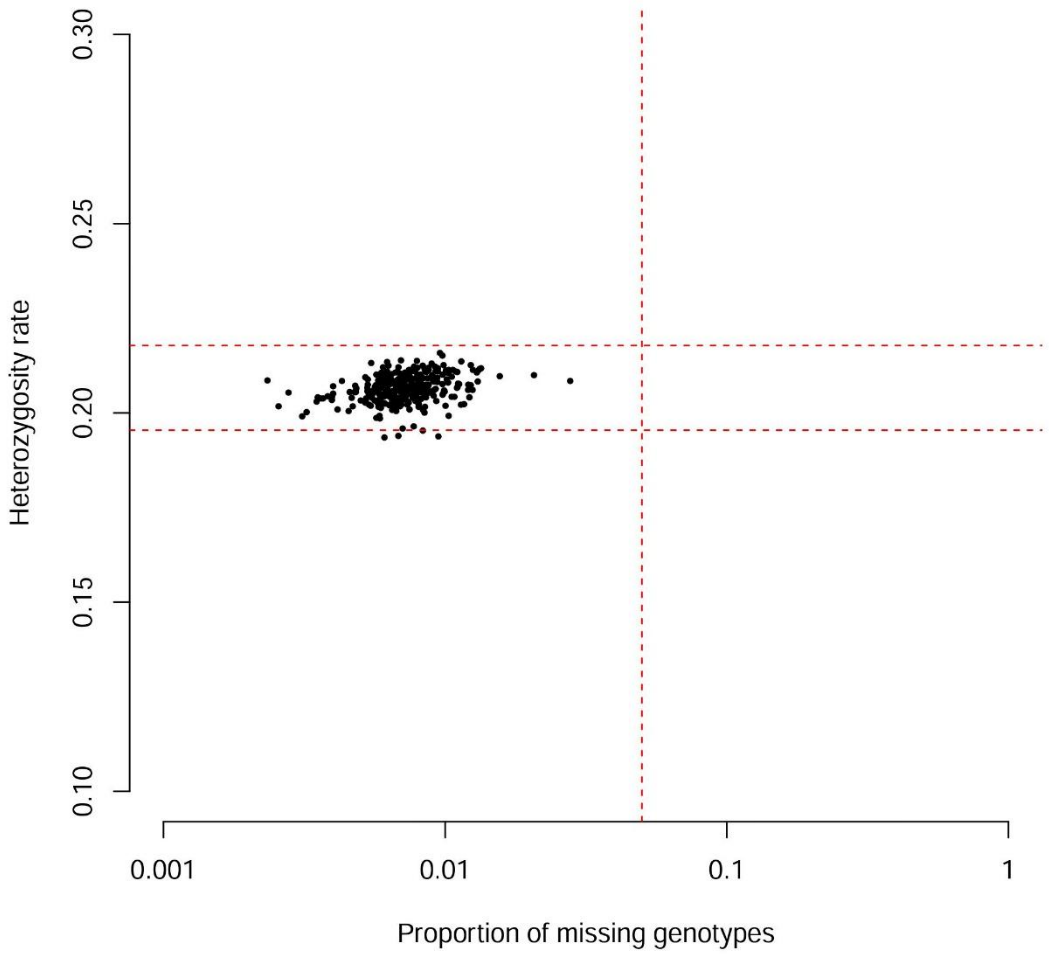

Supplemental Figure 3. The detection of identity by descent segments in close relative pairs using unphased dense SNP data shows pairwise relatedness in the cohort. MZ indicates monozygotic twins, FS indicates full-siblings, PO indicates parent-offspring, and HS indicates half-siblings.

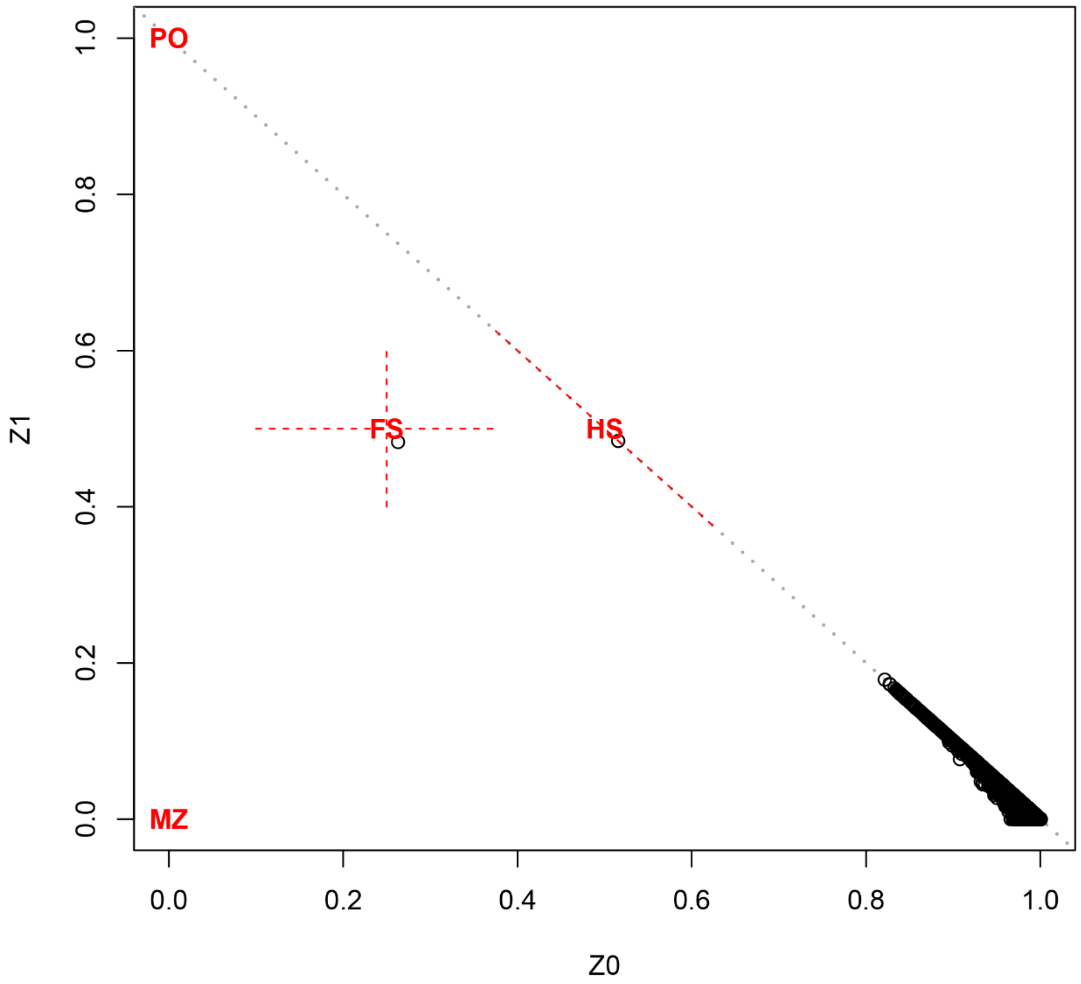

Supplementary Figure 4. Individuals were plotted based on the first two eigenvectors produced by principal component analysis (PCA), (A) based on the genotype data of 11 populations from HapMap (African ancestry in southwestern USA [ASW], Utah residents with northern and western European ancestry from the CEPH collection [CEU], Han Chinese in Beijing, China [CHB], Chinese in metropolitan Denver, Colorado [CHD], Gujarati Indians in Houston, Texas [GIH], Japanese in Tokyo, Japan [JPT], Luhya in Webuye, Kenya [LWK], Mexican ancestry in Los Angeles, California [MEX], Maasai in Kinyawa, Kenya [MKK], Tuscan in Italy [TSI], and Yoruban in Ibadan, Nigeria [YRI]) and from the cohort of the present study (HAN). (B) Based on the genotype data of our cohort in the present study.

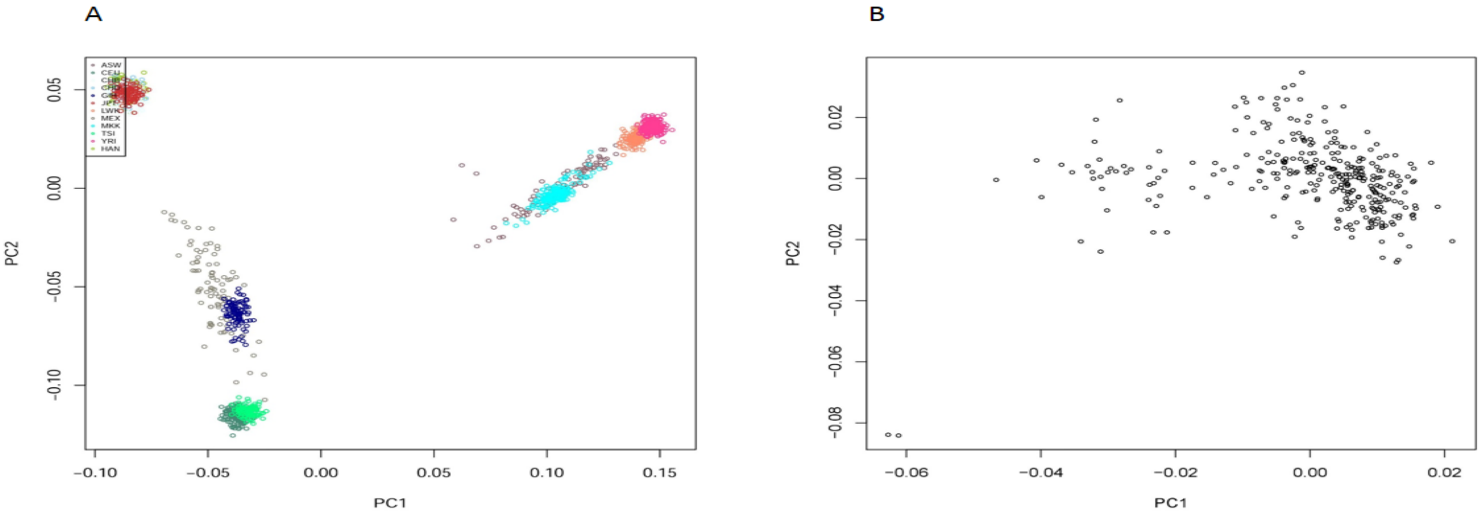

Supplemental Figure 5. Quantile-quantile plots for (A) linoleic acid, (B) α-linolenic acid (C) gamma-linolenic acid, (D) dihomo-gamma-linolenic acid, (E) arachidonic acid, (F) eicosapentaenoic acid, (G) adrenic acid, (H) docosapentaenoic acid, and (I) docosahexaenoic acid. The observed P-values of the indicated SNPs are plotted against the theoretical distributions of the expected P-values. A total of 32,387 SNPs were used to generate each plot.

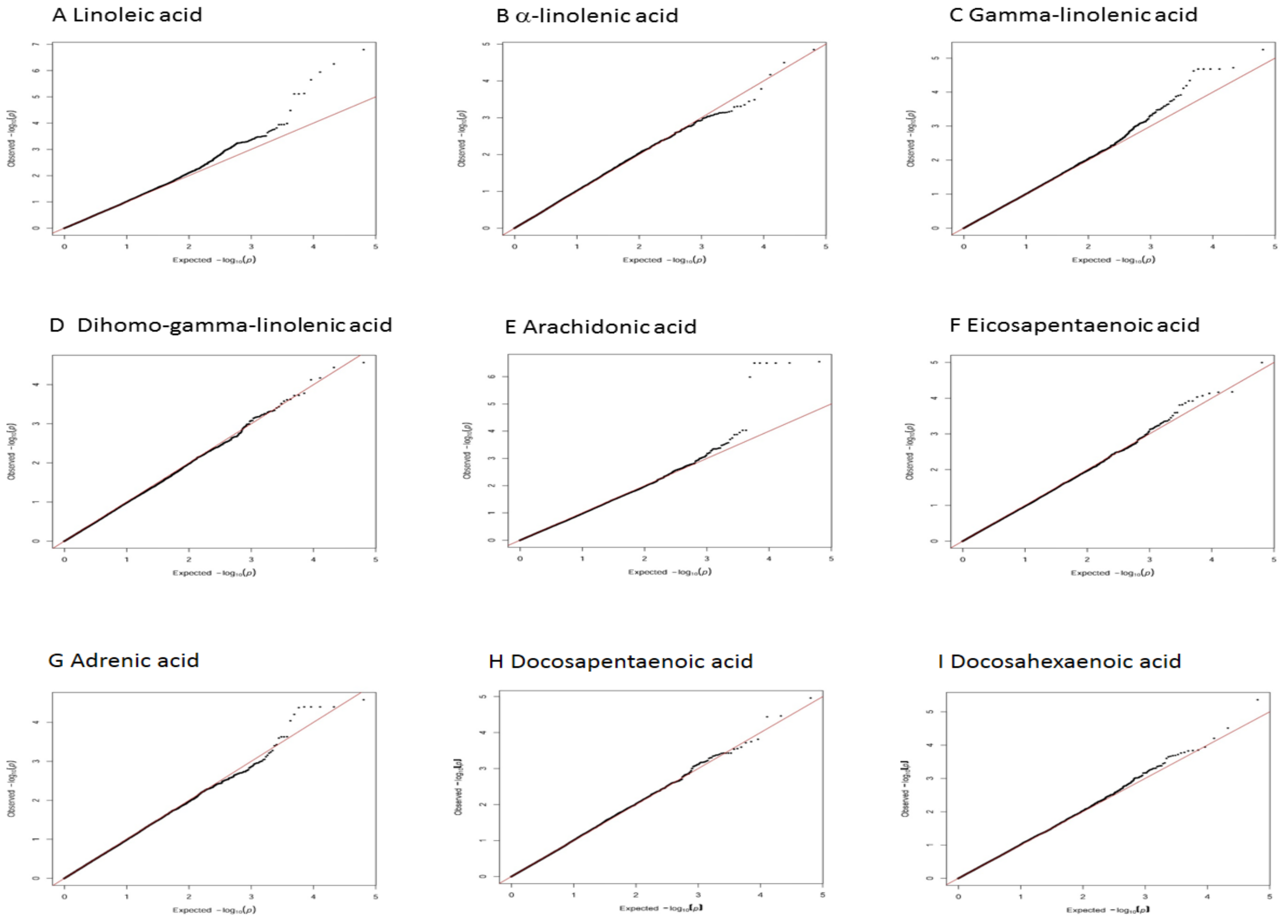

Supplemental Figure 6. Linkage disequilibrium maps for single nucleotide polymorphisms (SNPs) genotyped in *FADS* and the flanking regions. Color schemes are based on the D’ value in the Haploview software. The shades of red show the strength of the pairwise linkage disequilibrium, and the numbers represent the D’ values expressed as percentages.

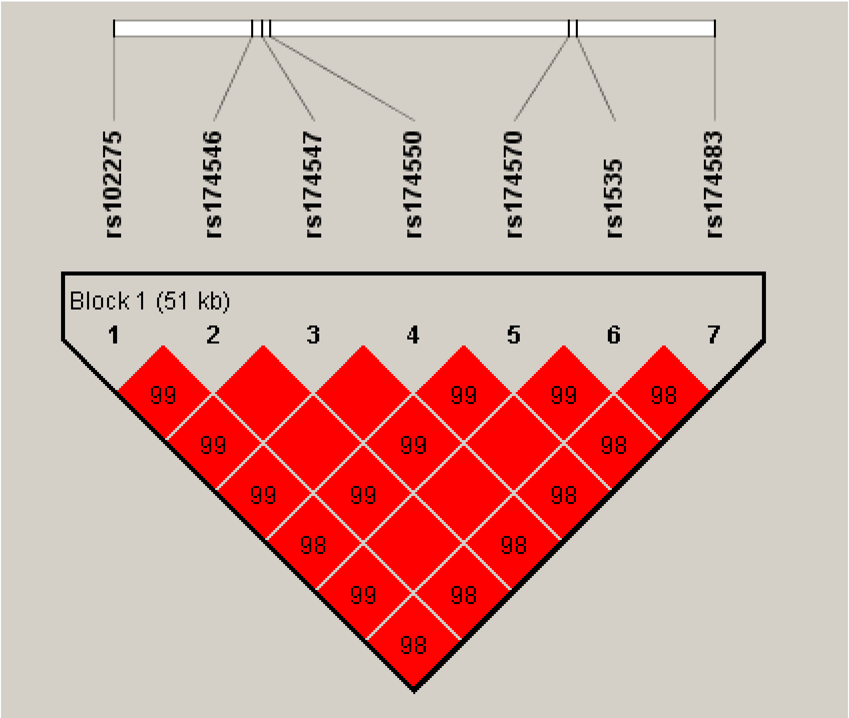

Supplement: Supplementary file 1 — Supplementary data. Table S1. Quality control (QC) at the individual level. Table S2. Quality control (QC) at the SNP level. Table S3. Characteristics of the seven SNPs analyzed in the C11orf10 /FADS1/FADS2 gene cluster. Table S4. Associations between rs174570 and free fatty acid levels. Figure S1. The participant flow chart in this study. SHOS, Shanghai Obesity Study; MH-NW, metabolically healthy participants with normal BMI; MHO, metabolically healthy participants with overweight or obese state; metabolically unhealthy participants with overweight or obese state. Figure S2. The proportion of missing genotypes against the heterozygosity rate is plotted. The overall call rate is greater than 0.98, and the heterozygosity rate is also considered to remove tainted and inbred samples. Figure S3. The detection of identity by descent segments in close relative pairs using unphased dense SNP data shows pairwise relatedness in the cohort. MZ indicates monozygotic twins, FS indicates full-siblings, PO indicates parent-offspring, and HS indicates half-siblings. Figure S4. Individuals were plotted based on the first two eigenvectors produced by principal component analysis (PCA), (A) based on the genotype data of 11 populations from HapMap (African ancestry in southwestern USA [ASW], Utah residents with northern and western European ancestry from the CEPH collection [CEU], Han Chinese in Beijing, China [CHB], Chinese in metropolitan Denver, Colorado [CHD], Gujarati Indians in Houston, Texas [GIH], Japanese in Tokyo, Japan [JPT], Luhya in Webuye, Kenya [LWK], Mexican ancestry in Los Angeles, California [MEX], Maasai in Kinyawa, Kenya [MKK], Tuscan in Italy [TSI], and Yoruban in Ibadan, Nigeria [YRI]) and from the cohort of the present study (HAN). (B) Based on the genotype data of our cohort in the present study. Figure S5. Quantile-quantile plots for (A) linoleic acid, (B) a-linolenic acid (C) gamma-linolenic acid, (D) dihomo-gamma-linolenic acid, (E) arachidonic acid, (F) eic [file 13148_2018_545_MOESM1_ESM.pdf]
